# Differing metabolic responses to salt stress in wheat-barley addition lines containing different 7H chromosomal fragments

**DOI:** 10.1371/journal.pone.0174170

**Published:** 2017-03-22

**Authors:** Eva Darko, Krisztián Gierczik, Orsolya Hudák, Péter Forgó, Magda Pál, Edina Türkösi, Viktória Kovács, Sándor Dulai, Imre Majláth, István Molnár, Tibor Janda, Márta Molnár-Láng

**Affiliations:** 1 Department of Plant Physiology, Agricultural Institute, Centre for Agricultural Research, Hungarian Academy of Sciences, Martonvásár, Hungary; 2 Department of Plant Molecular Biology, Agricultural Institute, Centre for Agricultural Research, Hungarian Academy of Sciences, Martonvásár, Hungary; 3 Food and Wine Knowledge Centre, Faculty of Science, Eszterházy University, Eger, Hungary; 4 Department of Genetic Resources, Agricultural Institute, Centre for Agricultural Research, Hungarian Academy of Sciences, Martonásár, Hungary; 5 Department of Botany and Plant Physiology, Faculty of Science, Eszterházy University, Eger, Hungary; Institute of Genetics and Developmental Biology Chinese Academy of Sciences, CHINA

## Abstract

Salinity-induced osmotic, ionic and oxidative stress responses were investigated on Asakaze/Manas wheat/barley addition lines 7H, 7HL and 7HS, together with their barley (salt-tolerant) and wheat (relatively salt-sensitive) parents. Growth, photosynthetic activity, chlorophyll degradation, proline, glycine betaine accumulation, sugar metabolism, Na^+^ and K^+^ uptake and transport processes and the role of polyamines and antioxidants were studied in young plants grown in hydroponic culture with or without salt treatment. Changes in plant growth and photosynthetic activity of plants demonstrated that the salt tolerance of the addition lines 7H and 7HL was similar to that of barley parent cv. Manas, while the sensitivity of the addition line 7HS was similar to that of the wheat parent cv. Asakaze. The Na accumulation in the roots and shoots did not differ between the addition lines and wheat parent. The activation of various genes related to Na uptake and transport was not correlated with the salt tolerance of the genotypes. These results indicated that the direct regulation of Na transport processes is not the main reason for the salt tolerance of these genotypes. Salt treatment induced a complex metabolic rearrangement in both the roots and shoots of all the genotypes. Elevated proline accumulation in the roots and enhanced sugar metabolism in the shoots were found to be important for salt tolerance in the 7H and 7HL addition lines and in barley cv. Manas. In wheat cv. Asakaze and the 7HS addition line the polyamine metabolism was activated. It seems that osmotic adjustment is a more important process in the improvement of salt tolerance in 7H addition lines than the direct regulation of Na transport processes or antioxidant defence.

## Introduction

Salt accumulation in the soil is one of the most important abiotic stresses limiting plant growth and causes significant yield losses throughout the world. Salinity induces osmotic, ionic and oxidative stress in cells.

Osmotic stress, provoked even by relatively moderately salinity levels, decreases soil water potential, reduces water uptake and may cause cell dehydration, leading to limited cell elongation, stomatal closure and a decline in carbon assimilation. By causing ionic stress, excess NaCl disturbs the integrity and selectivity of plasma membranes and modifies the uptake and homeostasis of essential ions. Intense salt stress alters several enzymatic and metabolic processes, which may also contribute to a decline in photosynthesis and accelerated chlorophyll degradation. The accumulation of reactive oxygen species (ROS) triggered by salt stress stimulates metabolic responses, but excess ROS may also cause secondary stress to plants [1]. Large-scale proteomic, metabolomic and transcriptomic analyses revealed that salt stress causes a complete metabolic rearrangement in cells, affecting the sugar, amino acid and polyamine metabolism, and ion and redox homeostasis [2–5]. However, these responses show tissue specificity and depend greatly on the genotype and species, salt concentration, length of exposure, stage of plant development and environmental conditions.

Salt tolerance mechanisms involve multiple processes, including the exclusion of Na^+^ from the cytosol by regulating Na^+^ uptake and transport mechanisms in different parts of the roots and shoots and/or by the compartmentation of Na^+^ into the vacuoles. Various transporters, such as SOS1 (Salt Overly Sensitive), a plasma membrane Na^+^/H^+^ exchanger together with the SOS2 and SOS3 signalling proteins, high-affinity K^+^ transporters (HKT1), tonoplast Na^+^/H^+^ antiporters (NHX) and H^+^-pyrophosphatase (HVP), are reported to play a fundamental role in these processes (see review in [6,7]). In addition, several types of compatible solutes, such as N-containing compounds (e.g. proline, glycine betaine), sugars (e.g. sucrose, trehalose, fructans), and various polyols may accumulate in the cytoplasm as osmoprotectants to alleviate salt stress. Furthermore, the activation of the general defence systems, which involves not only antioxidant compounds and enzymes, but also polyamines (PAs) such as putrescine (PUT), spermidine (SPD) and spermine (SPN), may help to maintain the ion and redox homeostasis of the cell [8–10].

In spite of wide knowledge on both the effect of salt and the mechanisms of salt tolerance, there is no general conclusion on crop strategies for improving salt tolerance. Any of these mechanisms or their combination may be responsible for the salt tolerance of plant genotypes and species [4,11].

Among the cereals, barley (*Hordeum vulgare* L.) is regarded as a more salt-tolerant crop than cultivated wheat (*Triticum aestivum* L.) [5,12]. This is also true of the barley cv. Manas. When the salt tolerance of barley cv. Manas was compared to that of several wheat lines (Chinese Spring, Asakaze and Mv9kr1), Manas was found to retain higher germination potential, growth ability and photosynthetic activity under salt stress conditions than the wheat genotypes, including Asakaze, although this cultivar is moderately salt-tolerant [13,14]. Both the Na^+^ accumulation process and the osmotic adjustment capacity of Manas differed from those of the wheat genotypes [14]. In addition, studies on the salt stress response of Asakaze/Manas wheat/barley addition lines (2H-7H) revealed that elevated salt tolerance can also be observed in the 7H disomic addition line. Using ditelosomic lines, containing only the short or long arm of the 7H chromosome (7HS and 7HL), the 7HL ditelosomic line was found to have a similar phenotype and photosynthetic activity as 7H and barley cv. Manas [14,15], while the 7HS ditelosomic line had low germination potential under salt stress conditions, like that of wheat cv. Asakaze [16].

In spite of these initial successes, no detailed investigation has yet been made to reveal the metabolic changes responsible for the difference in salt stress response of these Asakaze/Manas addition lines. To achieve this goal, several metabolic processes related to the synthesis of osmolytes, ion transport, signalling and redox balance were investigated. This paper compares the results obtained for proline and glycine betaine (GB) accumulation, sugar composition and metabolism, Na^+^ and K^+^ uptake and transport processes, and the role of polyamines and antioxidants in the wheat/barley 7H disomic and 7HL and 7HS ditelosomic addition lines and in the wheat and barley parents. The use of these lines together with their wheat and barley parental genotypes made it possible to study the separate effects of the short and long arms of chromosome 7H on salinity-induced metabolic changes.

## Materials and methods

### Plant material and experimental design

The investigations were performed on a Japanese facultative bread wheat cv. Asakaze, a Ukrainian six-rowed winter barley cv. Manas, and the 7H disomic addition line and 7HL and 7HS ditelosomic addition lines developed from the Asakaze/Manas hybrid as described in [16,17]. The genetic stability of the addition lines was checked cytologically several times [16]. The 7HS and 7HL ditelosomic addition lines contain only the short (7HS) or long (7HL) arm of the 7H chromosome, as presented in S1 Fig.

The experimental design and hydroponic growth conditions were similar to those described in [14]. Briefly, 3-day-old germinated seedlings with similar root length were grown in pots (10 plants/0.6L) containing modified Hoagland solution [18] in a phytotron growth chamber (PGR 15, Conviron, Controlled Environments Ltd., Winnipeg, MB, Canada) under a 16h photoperiod at 120 μmol m^-2^ s^-1^ and with 22/20°C day/night temperature. Salt stress was induced in 7-day-old plants by applying increasing concentrations of NaCl (100, then 200mM) in seven-day cycles. The control plants continued to grow without salt treatment. The solutions were renewed every two days.

### Monitoring plant growth and biomass production of plants

To follow the salt-induced decrease in growth, the root and shoot length were measured before and after the salt treatments. The biomass production of the lines was determined by measuring the root and shoot weight of the plants at the end of the experiments.

### Measurements of plant CO_2_ assimilation and chlorophyll content

The photosynthetic activity and chlorophyll content of the plants were determined to monitor their salt-induced physiological responses. A Ciras 2 portable photosynthesis system with a narrow (2.5 cm^2^) leaf cuvette (Amesbury, USA) was used to measure the net photosynthetic rate (Pn), stomatal conductance (gs), transpiration rate (E) and intracellular CO_2_ concentration (Ci). These parameters were determined on the 3^rd^ fully expanded leaves at the steady-state level of photosynthesis using a CO_2_ level of 380 μL L^-1^ and light intensity of 750 μmol m^-2^ s^-1^.

The chlorophyll content of intact leaves was estimated at the end of the experiments using a SPAD-502 chlorophyll meter (Spectrum Technologies, Plainfield, IL, USA).

### Determination of Na and K content in leaf and root

The amounts of sodium and potassium were determined from air-dried leaf samples (0.5g/sample) using the inductively coupled plasma-atomic emission spectrometry method (ICP-AES, Jobin-Yvon Ultima 2 sequential instrument) after microwave Teflon bomb digestion with cc. HNO_3_+HCl [19]. Three samples of each genotype and treatment were collected for analysis at the end of the experiments.

### Gene expression studies

The gene expression levels of several genes responsible for Na^+^ transport and sequestration were determined by quantitative reverse transcription PCR (qRT-PCR). For this study, root and leaf samples were collected at the end of the experiment in three biological replicates originating from different pots of each genotype and treatment. The total RNA content was isolated with a Direct-zol^™^ RNA MiniPrep Kit (Zymo Research, USA) and TRI-Reagent (Zymo Research, USA) according to the manufacturer's instructions. M-MLV-Reverse transcriptase was used for the cDNA synthesis as described in [20].

Quantitative RT PCR measurements were performed with the CFX96 Touch^™^ Real-Time PCR Detection System (Bio-Rad Hungary Ltd., Hungary) using the KAPA SYBR^®^ FAST, Master Mix (2X), Universal qPCR Kit (Kapa Biosystems, Inc., Wilmington, USA). Primers (S1 Table) for the genes *SOS1*, *SOS2*, *HKT1*, *NHX2* and *HVP1* were either designed with the NCBI—Primer Design Tool software (National Center for Biotechnology Information, Bethesda, USA) or the sequences were taken from the literature [21,22]. Several reference genes suitable for both wheat and barley genotypes were tested at the suggestion of Giménez *et al*. [23]. Although the *CDC(a)* and *RLI(a)* genes operated both in wheat and barley, the Ct values of these reference genes were found to shift significantly under salt stress conditions (S2 Fig), making them unsuitable as reference genes. Consequently, as proposed by Burton *et al*. [21] and Paolacci *et al*. [22], the conserved *cyclophilin* gene was used as the reference gene for barley and the *Ta30797* gene for wheat and the addition lines, as these only showed a shift in Ct value of 1.0 and 0.8, respectively, under salt stress. For each reaction a melt curve was determined to confirm the amplification of a single gene product. The efficiency values were between 90 and 100% for all the primers, so relative transcript levels were calculated with the ΔΔCt method [24].

### Determination of proline, glycine-betaine, soluble sugar and starch content

The proline, glycine-betaine (GB) and total soluble sugar contents of the samples were determined photometrically using a UV-Visible spectrophotometer (160A, Shimadzu Corp, Kyoto, Japan). The proline content was determined on the basis of its reaction with ninhydrin, according to the Bates method [25] as described in [26]. The GB content was determined using the periodide method [27] as described in [28] and the soluble sugars were estimated by the anthrone reagent method, as described in [26].

The megazyme total starch assay protocol was used to determine the starch content of the samples (K-TSTA-100A, Megazyme International Ireland, Wicklow, Ireland). The method is based on the enzymatic hydrolysis of starch to glucose using thermostable α-amylase and amyloglucosidase. The starch content was determined from the twice-washed pellet remaining after the extraction of total soluble sugars.

For each method, 5 x 0.2 g fresh weight (FW) samples were collected at the end of the experiments. Standards ranging from 0–50 μg/mL for the proline calibration curve, 0–400 μg/mL for GB and 0–100 μg/mL for glucose calibration curves were used to determine the metabolite contents of the samples, expressed in μg/g FW.

### Determination of soluble sugars in leaves with HPLC-ELS

The soluble sugar composition was determined with the HPLC method using evaporative light scattering (ELS) detection. The leaf samples were extracted as described for total soluble sugars and the supernatants were filtered through a 0.45μm membrane filter (ProFill-RC) for the HPLC analysis. The chromatographic experiments were set up using a Shimadzu LCMS2010EV instrument with a Prevail carbohydrate column (250 × 4.6 mm, 5 μm) equipped with a guard column. A binary eluent system involving water (A) and acetonitrile (B) was used in a gradient setup: t[minutes](B%):0(70)-20(60)-110(40)-120(0)-130(0)-140(70)-150(70)]. The samples (2–10 μL) were injected into a 0.8 mL/min eluent flow. A PL-ELS-2100 evaporative light scattering detector (Polymer Laboratories) was used to detect the carbohydrate signals; the evaporation temperature was 90°C, the nebulization temperature 50°C and the nitrogen gas flow rate 1.6 l/min. The chromatographic signals were integrated manually or using the splitting integral mode of the processing software. Low-molecular-weight standards (glucose, fructose, sucrose and maltose) were used to calculate the amounts of sugars. The amount of unknown sugars was estimated on the basis of their relative percentage values and calculated as glucose equivalent.

### Determination of polyamine content by HPLC

Polyamine analysis was carried out from leaf and root samples collected at the end of the experiments according to the modified protocols of Smith and Davies [29]. Briefly, 0.2 g samples were extracted in 1mL of 0.2 M ice-cold perchloric acid and stored for 20 min on ice. After centrifugation for 10 min at 10 000g the supernatant was used for the determination of free and conjugated polyamines, namely PUT, SPD and SPN. Dansylated polyamines were separated by HPLC using a W2690 separation module on a reverse phase column (Kinetex C18, 5μ, 100 x 4.6 mm, Phenomenex, Inc.) and detected with a W474 scanning fluorescence detector with excitation at 340 nm and emission at 515 nm. Conjugated forms of PAs were measured after acid hydrolysis at 96°C for 1h. Three samples were collected from each genotype and treatment for this analysis.

### Determination of the activities of antioxidant enzymes and invertase

Spectrophotometric methods were used to determine the activity of the antioxidant enzymes ascorbate peroxidase (APX), monodehydroascorbate reductase (MDHAR), catalase, glutathione reductase (GR) and glutathione-S-transferase (GST) and to measure the activity of the invertase (β-d-fructofuranosidase fructohydrolase) enzyme. The sample preparation and enzyme assays for antioxidant enzymes and invertase were carried out as described in [30] and [31], respectively. For each type of measurement, leaf and root samples (5x 0.3 g) were collected at the end of the experiments and the activities were expressed as μmol substrate g^-1^ FW min^-1^.

### Statistics

The results were obtained from 3 independent experiments with 5 pots of each line per treatment (5 pots with and 5 pots without salt treatment). Samples were collected from each pot and the measurements were performed on 3–5 biological replicates. The SPSS 16.0 statistical program and Tukey’s *post hoc* test were used to determine differences between treatments and genotypes. Different letters indicate significant differences at the P < 0.05 level. The genotypes and the effects of salt treatment were also compared as D = [(Genotype − Asakaze)/Genotype*100] and [(Salt-treated − Control)/Salt-treated*100] for each measured parameter.

## Results

### Effect of salt stress on plant growth and photosynthetic activity

The harmful effect of salt stress was followed by monitoring the root and shoot growth and by determining the photosynthetic activity and chlorophyll content of the plants.

**Fig 1:**
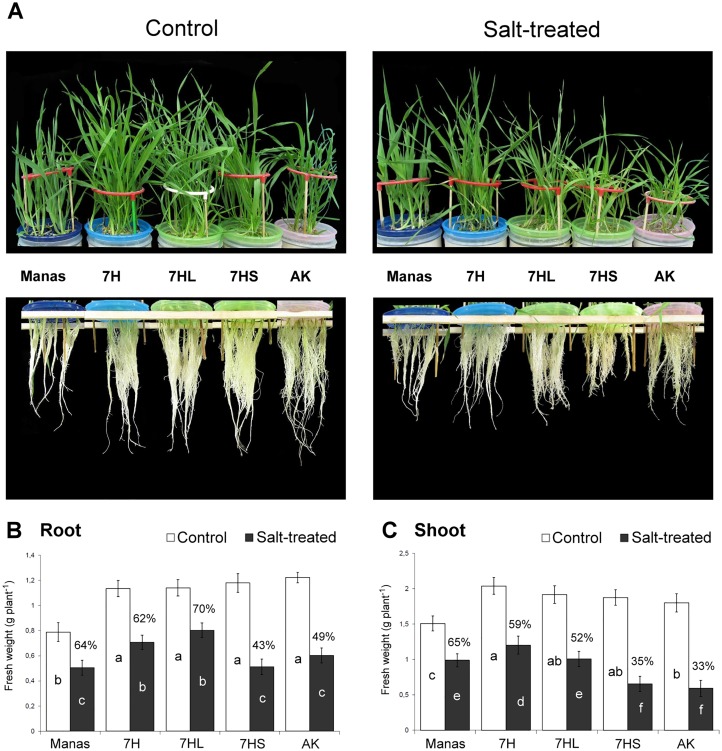
Growth responses of different genotypes grown in hydroponic solution with or without salt treatment. Typical plant phenotypes (A), and the root (B) and shoot (C) weights determined at the end of the experiments. Data are means ± SD of 25 replicates per treatment and genotype. Salt-induced growth reduction was indicated as a % of the control. Different letters indicate significant differences between the genotypes at P < 0.05 using Tukey’s *post hoc* test.

Fig 1 shows plants of individual genotypes grown in hydroponic solution with or without salt application, and demonstrates the typical symptoms of salt stress, such as growth reduction and chlorosis. Comparing the genotypes, the lowest root and shoot lengths were found for barley cv. Manas, while wheat cv. Asakaze and addition lines 7H, 7HL and 7HS had similar growth without stress conditions (Fig 1 and S2 Table). Salt treatment inhibited the root and shoot growth of Asakaze and addition line 7HS more intensively than that of addition lines 7H and 7HL or Manas (S2 Table and Fig 1B and 1C). This was manifested in the salt-induced decrease in the weight and length of the roots and shoots. At the end of the experiments, the weight and length of the roots and shoots ranged from 52–77% of the control values in the 7H and 7HL addition lines and Manas, and from 20–49% for addition line 7HS and Asakaze (S2 Table, Fig 1B and 1C), demonstrating their different sensitivity to salt.

Salt stress induces rapid stomatal closure and a decrease in the photosynthetic activity. The decline in photosynthetic activity (Pn) and stomatal conductance (gs) was more pronounced in wheat cv. Asakaze and addition line 7HS than in addition lines 7H and 7HL and in Manas (S3 Table). The salt-induced decrease in the chlorophyll content of the leaves was also greater in Asakaze and addition line 7HS than in the other genotypes (Fig 1 and S3 Table). These results indicated that Manas and addition lines 7H and 7HL were better able to maintain their growth potential and photosynthetic activity under salt stress conditions than Asakaze and addition line 7HS.

Several metabolic processes were then investigated to reveal which of them may be responsible for differences in the salt stress response of these lines.

### Na^+^ and K^+^ accumulation and transport in plants, and the expression of salt-responsive genes

**Fig 2:**
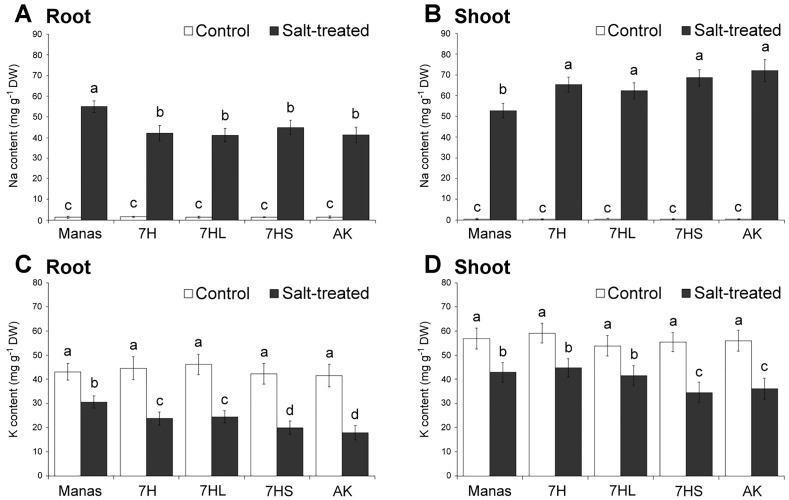
Na^+^ (A,B) and K^+^ (C,D) contents of roots and shoots of different genotypes (wheat/barley disomic addition line 7H, ditelosomic addition lines 7HL and 7HS, wheat cv. Asakaze: AK and barley cv. Manas) grown in hydroponic solution with or without salt treatment. Values were determined from air-dry samples collected at the end of the experiments, and data are means ± SD of three replicates per treatment. Different letters indicate significant differences between the genotypes at P < 0.05 using Tukey’s *post hoc* test.

The Na^+^ and K^+^ contents were determined from root and shoot samples of each genotype grown under control and salt-stress conditions (Fig 2). Salt stress induced considerable Na^+^ accumulation while inhibiting K^+^ uptake and transport in both the roots and leaves. No substantial difference was observed in the Na^+^ and K^+^ contents of the various control plants, while their response to salt stress differed. A slightly higher level of Na^+^ accumulation was observed in the roots of barley cv. Manas and a lower level in the shoots as compared to the other genotypes, indicating the restricted transport of Na^+^ from the roots to the shoots in Manas. A comparison of the genotypes revealed that the Na^+^ level in the roots of the disomic addition line 7H and of 7HL and 7HS was similar to that of wheat cv. Asakaze, while that in the shoots was closer to that of the wheat parent cv. Asakaze than the barley parent cv. Manas. This suggests that Na accumulation was not modified substantially in the addition lines (Fig 2A and 2B). Salt stress inhibited the K^+^ uptake and transport both in the roots and shoots (Fig 2C and 2D). However, higher K^+^ content was retained in the roots and shoots of barley cv. Manas than in the other genotypes. Higher K^+^ content was also observed in the 7H and 7HL addition lines than in wheat cv. Asakaze, while the 7HS genotype showed values similar to that of the wheat parent (Fig 2C and 2D). The different K contents of the genotypes were also manifested in the K/Na ratio (S3 Fig). The K/Na ratio was higher in Manas, 7H and 7HL both in the roots and shoots than in Asakaze and the 7HS addition line. Since the Na^+^ content of the 7H and 7HL addition lines was similar to that of wheat, it seems that the higher K/Na ratio was due to better K retention.

**Fig 3:**
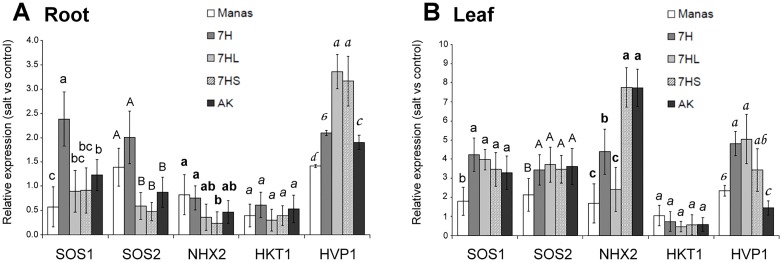
Salt-induced changes in the expression levels of the *SOS1* (*salt-overly sensitive 1*), *SOS2 (salt-overly sensitive 2*), *NHX2* (Na/H antiporter), *HVP* and *HKT1* (histidine-kinase transporter) genes in the roots (A) and leaves (B) of barley cv. Manas, wheat/barley disomic addition line 7H, ditelosomic addition lines 7HL and 7HS and wheat cv. Asakaze (AK). The data originated from three biological and three technical replicates of each treatment. For each gene, different letters indicate statistically significant differences between the genotypes at P < 0.05 using Tukey’s *post hoc* test.

The expression levels of several key genes potentially involved in Na^+^ uptake, transport and sequestration (*SOS1*, *SOS2*, *HKT1*, *NHX2* and *HVP1)* were investigated (Fig 3). Surprisingly, the transcript level of these genes did not increase so intensively in salt-treated roots as in salt-treated leaves. In the roots, the expression of the *HVP1* gene was higher in ditelosomic lines 7HL and 7HS than in the other genotypes. In the roots, the expression of the *SOS1* gene was slightly higher in the 7H line, and that of *SOS2* in 7H and Manas compared to the other genotypes. The expression of *NHX2* and *HKT1* was low in the roots of all the genotypes (Fig 3A).

In the leaves, the expression of the *SOS1* and *SOS2* genes was lower in barley cv. Manas than in the other genotypes, but no significant difference was observed between the addition lines and wheat cv. Asakaze (Fig 3B). The transcript level of the *HVP1* gene increased especially in addition line 7H and ditelosomic lines 7HL and 7HS, and was also higher in Manas than in Asakaze. The highest level of gene expression was detected for *NHX2* in the leaves of wheat cv. Asakaze and the 7HS ditelosomic line, differing substantially from that in 7H, 7HL and Manas. The expression level of the *HKT1* gene was low in the leaves of all the genotypes.

### Accumulation of proline and glycine–betaine

Previous results demonstrated that osmotic adjustment may play an important role in the maintenance of growth and photosynthetic activity under salt stress in these genotypes [14]. Measurements of osmotic potential were repeated, and revealed a similar tendency to that found previously [14] (data not shown). In addition, the osmotic adjustment of addition line 7HS resembled that of the wheat parent Asakaze (data not shown).

**Fig 4:**
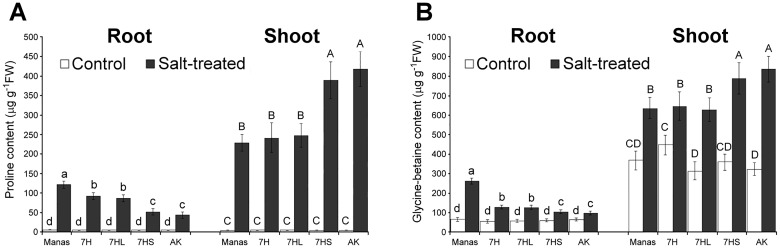
Proline (A) and glycine-betaine (B) contents of roots and shoots of different genotypes (wheat/barley disomic addition line 7H, ditelosomic addition lines 7HL and 7HS, wheat cv. Asakaze:AK and barley cv. Manas) grown in hydroponic solution with or without salt treatment. Data are means ± SD of five replicates per treatment. Different letters indicate significant differences between the genotypes at P < 0.05 using Tukey’s *post hoc* test.

In the present study, several metabolites typically accumulated during salt stress were studied, including proline and glycine-betaine (GB), together with compounds involved in the sugar metabolism. The contents of both the proline and glycine-betaine were low in root and shoot samples of untreated plants, but increased significantly during salt stress (Fig 4A and 4B). In the roots, higher proline and GB contents were observed in barley cv. Manas and in the 7H and 7HL addition lines than in wheat cv. Asakaze and the 7HS line. In contrast, the proline and GB accumulation was significantly lower in the leaves of Manas and the 7H and 7HL additions than in Asakaze and the 7HS genotype. This indicates that different mechanisms operate in the roots and shoots. These mechanisms may be related to the salt tolerance of the genotypes, as barley the parental line and the 7H and 7HL additions had better salt tolerance than Asakaze or 7HS.

### Sugar metabolism in plants

**Fig 5:**
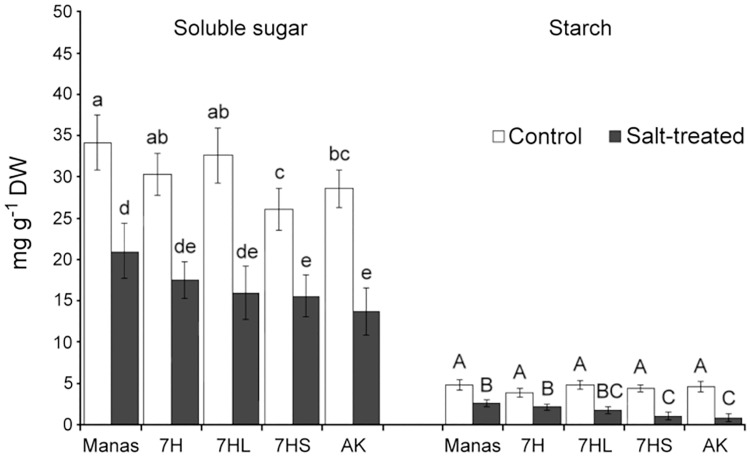
Total soluble sugar (A) and starch (B) contents in the roots of different genotypes (wheat/barley disomic addition line 7H, ditelosomic addition lines 7HL and 7HS, wheat cv. Asakaze: AK and barley cv. Manas) grown in hydroponic solution with or without salt treatment. Data are means ± SD of five replicates per treatment. Different letters indicate significant differences between the genotypes at P < 0.05 using Tukey’s *post hoc* test.

The role of carbohydrates as osmoprotectants was also investigated. First, the total amounts of soluble sugars and starch were determined. In the roots both the sugar and starch contents were significantly lower than in the leaves, and their amount decreased by 40–50% in all genotypes after salt treatment (Fig 5). These results suggest that under the present experimental conditions sugar metabolites did not contribute significantly to the maintenance of osmotic potential in the roots under salt stress conditions.

**Fig 6:**
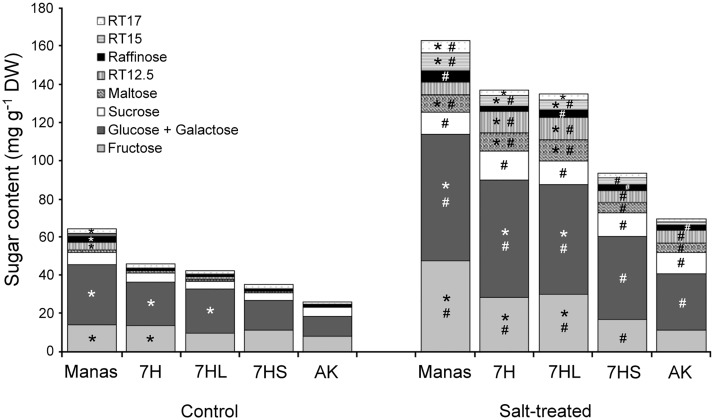
Sugar composition in the leaves of different genotypes (wheat/barley disomic addition line 7H, ditelosomic addition lines 7HL and 7HS, wheat cv. Asakaze: AK and barley cv. Manas) grown in hydroponic solution with or without salt treatment. Data are means of three replicates per treatment. * and # indicate values significantly different from the corresponding AK or untreated samples at the P < 0.05 level.

In the leaves, however, intense soluble sugar accumulation was observed after salt treatment in all the genotypes, being significantly higher in barley cv. Manas and addition lines 7H and 7HL than in wheat cv. Asakaze and the 7HS line (S4 Fig). Therefore, detailed HPLC analyses were performed to determine which soluble sugar metabolites accumulated in the leaves (Fig 6). Unfortunately, glucose and galactose, which differ only in the position of the hydroxyl group on C4, could not be separated using the HPLC-ELS system. Without salt stress, higher glucose+galactose content was observed in addition lines 7H and 7HL and in Manas than in Asakaze and addition line 7HS. The quantity of raffinose and two of the unidentified sugars (RT12.5 and 17) was also higher in Manas than in the other genotypes, though they were present in significantly lower quantities than the main soluble sugars: glucose+galactose, fructose and sucrose. Most of the sugar compounds were accumulated under salt stress conditions in all the plants, while the relative proportion (%) of maltose and the sugars found at 12.5 and 15 minutes (RT12.5 and RT15) increased significantly in response to salt stress. Comparing the genotypes under salt stress conditions, higher fructose, glucose+galactose and maltose quantities were found in addition lines 7H and 7HL and barley cv. Manas than in wheat cv. Asakaze. In addition, the peaks of the unidentified sugars (RT12.5 and RT15) were also higher in addition lines 7H and 7HL and in Manas than in Asakaze or addition line 7HS.

**Fig 7:**
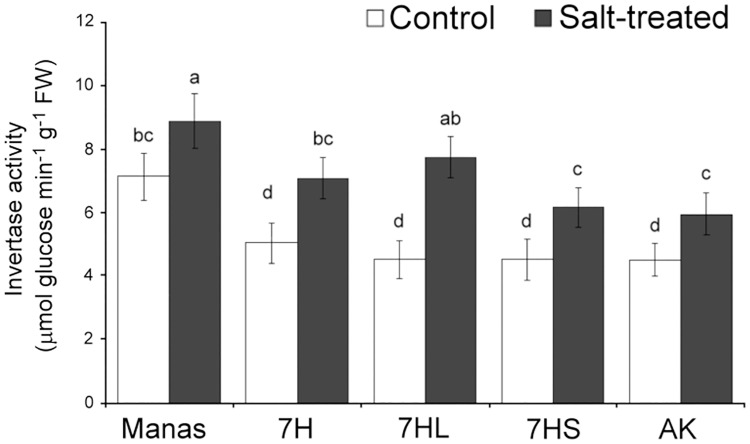
Invertase activity in control and salt-treated leaves of wheat/barley disomic addition line 7H, ditelosomic addition lines 7HL and 7HS, wheat cv. Asakaze: AK and barley cv. Manas. Data are means ± SD of five replicates per treatment. Different letters indicate significant differences between the genotypes at P < 0.05 using Tukey’s *post hoc* test.

Greater invertase activity, converting sucrose to fructose and glucose may be responsible for the elevated fructose and glucose contents, so the activity of this enzyme was also determined. As shown in Fig 7, the invertase activity was higher in Manas, 7H and 7HL under salt stress conditions than in Asakaze or addition line 7HS, so this could be partially responsible for the greater osmotic capacity of these genotypes. However, the salt-induced increase in the glucose+galactose peak was significantly higher than for fructose (which has the same molecular weight) under salt stress conditions, suggesting that the increase in glucose+galactose was only partially due to the increased activity of the invertase enzyme. It could also originate from the *de novo* synthesis of assimilates (such as glucose or galactose) due to elevated CO_2_ assimilation capacity.

**Fig 8:**
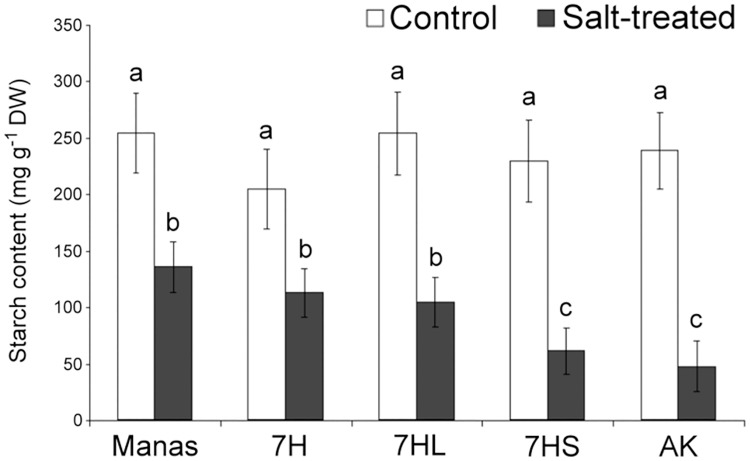
Starch content in control and salt-treated leaves of wheat/barley disomic addition line 7H, ditelosomic addition lines 7HL and 7HS, wheat cv. Asakaze: AK and barley cv. Manas. Data are means ± SD of five replicates per treatment. Different letters indicate significant differences between the genotypes at P < 0.05 using Tukey’s *post hoc* test.

The starch content was significantly lower in salt-treated plants than in control plants (Fig 8). Comparing the genotypes, the starch content was significantly higher in the barley genotype and in addition lines 7H and 7HL than in the wheat parent or addition line 7HS. These results indicate that starch degradation could have contributed to the increase in maltose in the leaves, but it could not be the main reason for the more intense sugar metabolism in salt-tolerant plants (barley cv. Manas, 7H and 7HL addition lines).

### Polyamine content and antioxidant enzyme activity in plants

**Fig 9:**
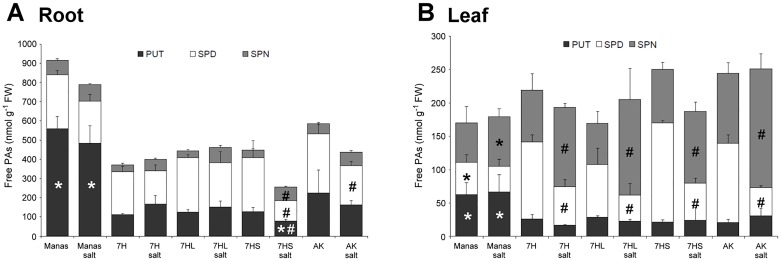
Free Polyamine (PA) content in the roots and shoots of different genotypes (wheat/barley disomic addition line 7H, ditelosomic addition lines 7HL and 7HS, wheat cv. Asakaze: AK and barley cv. Manas) grown in hydroponic solution with and without salt treatment. Data are means ± SD of three replicates per treatment. Data are mean of three replicates of each treatment. * and # indicate values significantly different from the corresponding AK or untreated samples at P < 0.05 level.

Polyamines (PAs), such as putrescine (PUT), spermidine (SPD) and spermine (SPN), have been reported to play a role in the abiotic stress response, including salt stress. However, this role may be complex, as they may function in the maintenance of ion homeostasis, as osmoprotectants and also in oxidative defence [10,32,33]. The determination of free (Fig 9) and conjugated (S5 Fig) PAs revealed that Manas contained significantly more PUT both in the roots and leaves than the other genotypes. Salt stress resulted in a decrease in PAs, especially SPD, in the roots of Asakaze and addition line 7HS, while there was hardly any change in Manas and addition lines 7H and 7HL. In the leaves, which contained significantly lower amounts of PAs than the roots, the SPD content decreased and SPN content increased intensively, especially in wheat cv. Asakaze and addition line 7HS, but the total amount of free and conjugated PAs (PUT, SPD and SPN) did not change after salt stress in most of the genotypes (except for a decrease in addition line 7HS). These results indicate that PAs may be more important in the salt stress response than in salt tolerance.

Salt stress may also induce oxidative damage in cells, as a secondary stress, while antioxidants regulate the amount of reactive oxygen species and give protection against oxidative damage. The role of antioxidant enzymes in protection against salt-induced oxidative stress was therefore studied by measuring the activities of ascorbate peroxidase (APX), monodehydroascorbate reductase (MDHAR), catalase, glutathione reductase (GR) and glutathione-S-transferase (GST) (S4 Table).

Salt stress raised the GR, APX and catalase activities in the roots and the GR and catalase activities in the shoots. Among the antioxidant enzymes tested, the salt-induced increase was the highest in the case of catalase both in roots and shoots. Comparing the genotypes, the catalase activity was higher in the roots of barley cv. Manas and addition lines 7H and 7HL than in wheat Asakaze and the 7HS addition line. In the shoots, however, the difference in the catalase activities of the genotypes was not significant.

## Discussion

The salt stress response of wheat-barley addition lines 7H, 7HL and 7HS was studied together with that of the wheat cv. Asakaze and barley cv. Manas parents. The results obtained for plant growth (Fig 1, S2 Fig), photosynthetic parameters and chlorophyll content (S2 Table) were in agreement with previous findings [14,15] indicating that barley cv. Manas has higher salt tolerance than wheat cv. Asakaze and that the presence of chromosome 7H or 7HL provided elevated salt tolerance in a wheat background. The role of the long arm of chromosome 7H was also confirmed by testing both ditelosomic addition lines 7HL and 7HS, which showed that the 7HL addition had similar growth and photosynthetic parameters to the 7H addition line, while 7HS was as sensitive as wheat cv. Asakaze.

The first logical explanation for the improved salt tolerance of the addition lines 7H and 7HL could be the modification of Na^+^ uptake and transport mechanisms, as several genes in the *HKT* and *HVP* subfamilies were found to be localized on chromosome 7H of barley [7,34].

In spite of this, there was no clear difference either in the Na accumulation in the roots and shoots of the addition lines and wheat cv. Asakaze (Fig 2) or in the expression patterns of the genes tested (Fig 3). The salt-induced gene expression patterns of several genes related to Na^+^ uptake and transport, the sequestration of Na from the cytosol and/or compartmentation into the vacuole did not differ significantly between the genotypes (e.g. *NHX2*, *HKT1* in the roots, *SOS1*, *SOS2*, *HKT1* in the shoots) or the difference was not related to salt tolerance (e.g. *HVP1*). Only the *NHX2* gene was expressed to a greater extent in salt-sensitive genotypes, with higher values in wheat cv. Asakaze and the 7HS addition line than in genotypes with improved salt tolerance. As the main role of the *NHX2* gene appears to be the sequestration of Na^+^ to the vacuole, this suggests that Na^+^ accumulation in the vacuole in the leaves of salt-sensitive plants is not able to protect the cytosol against excess Na^+^.

There were significantly more transcripts of the *HVP* gene both in the roots of addition lines 7HL and 7HS and in the shoots of addition lines 7H, 7HL and 7HS. Two genes (*HVP1* and *HVP10*) with very similar sequences were found to be localized on barley chromosome 7HL and 7HS, respectively [7]. It is possible that the primers used for the *HVP* gene hybridized to both *HVP1* and *HVP10*, providing high expression in both 7HL and 7HS. Although this confirmed that *HVP* genes are located on chromosome 7H, it is unlikely that the elevated expression of *HVP* genes is responsible for differences in the salt stress response of the 7HS and 7HL genotypes.

As the activation of the various genes tested and the Na accumulation in the roots and shoot was not correlated with the salt tolerance of the genotypes, the direct regulation of Na transport processes may not be the main reason for the salt tolerance of these genotypes. The lack of strong correlation between ion accumulation and the expression level of these genes is possible, because apart from HKT1, which is responsible for the Na transport from the roots to the shoots [35], the other genes are mainly responsible for the compartmentisation of excess Na within the cells. Therefore, their expression level does not directly influence the total ion content of the individual organs.

The maintenance of K^+^ in the cytosol is at least as important as the elimination of excess Na^+^. As documented by Smethurst *et al*. [36] and Cuin *et al*. [37], an improvement in the K^+^ retention ability of the cells (in root and shoot tissues) may result in elevated salt tolerance, irrespective of the Na^+^ content of the tissues. In this connection, although a higher concentration of K^+^ was retained in both the roots and shoots of barley cv. Manas and addition lines 7H and 7HL under salt stress conditions than in wheat cv. Asakaze and addition line 7HS (Fig 2), this difference was not great enough to suggest that the retention of K^+^ could be the main reason for improved salt tolerance.

The prevention of salt-induced K^+^ loss not only depends on the function of transporters [12], such as H^+^/ and Ca_2_^+^/ATPases [38,39], but also involves several metabolites. For example, it is documented that polyamines may affect ion transport directly (e.g. spermine blocks the K^+^ and non-selective cation channels, KIRC and NSCCs, respectively) or indirectly by enhancing plasma membrane stability under salt stress conditions [40,41]. In addition, polyamines may have a role in the maintenance of redox homeostasis through the activation of ROS-scavenging enzymes (see review in [10]). In addition, as polycations, polyamines may also serve as osmoprotectors [33,42]. The role of polyamines in the salt stress response and salt tolerance is controversial, perhaps due to their complex function in cells.

Intense changes were found in the free and conjugated polyamine contents in the roots and in their composition in the shoots, especially in wheat cv. Asakaze and addition line 7HS, which are relatively salt-sensitive compared to barley cv. Manas and addition lines 7H and 7HL (Fig 9, S5 Fig). These changes were mainly due to a decrease in SPD content, which seemed to be converted to SPN in the leaves. A similar tendency was observed in barley and rice, when cultivars differing in salt tolerance were compared [9,43,44]. The fact that changes in PA compounds are more intensive in salt-sensitive plants indicates that the decrease in spermidine and increase in spermine are more important in the general response of plants to salinity than in salt tolerance. The PUT content was higher in both the roots and shoots of salt-tolerant barley cv. Manas than in wheat or the addition lines even in the control, while the PAs did not exhibit a significant change during salt stress (Fig 9, S5 Fig). Higher PUT content was also found in other barley and oat cultivars as compared to wheat genotypes even without stress conditions [45], but direct evidence for a correlation between the amount of PUT and salt stress tolerance is difficult to find [46]. Since the changes in SPD and SPN were more pronounced in salt-sensitive plants and the elevated PUT content of barley cv. Manas was not manifested in the addition lines, it seems that polyamines do not play a direct role in the development of salt tolerance in addition lines 7H and 7HL.

Several papers indicated that the accumulation of osmolytes is a basic mechanism in the development of salt tolerance, and in the case of barley osmotic tolerance seems to be more beneficial than improved ion exclusion [5,47]. The elevated osmotic adjustment capacity of barley cv. Manas and of addition lines 7H and 7HL was previously detected by measuring the osmolality of leaf sap [14]. However, no investigations have yet been made to determine which osmolytes participate in osmotic adjustment.

As expected, salt stress induced the intense accumulation of proline and GB in plants. The amounts of these compounds were significantly higher in the roots of barley cv. Manas and addition lines 7H and 7HL than in wheat cv. Asakaze and addition line 7HS, while the soluble sugar content of the roots decreased in all the genotypes (Figs 4 and 5). In the leaves, however, the proline and GB content was lower in Manas and lines 7H and 7HL, while the sugar accumulation was significantly higher in these lines than in Asakaze and line 7HS (Figs 4 and 6). These results indicate that the salt tolerance of these genotypes may be detrimentally influenced by proline and GB in the roots and by the sugar metabolism in the shoots, and that different osmotic adjustment mechanisms may operate in the roots and shoots. This was also proved by Widodo *et al*. [2], who demonstrated that different metabolites accumulated in salt- tolerant and sensitive barley cultivars. Sugar metabolites increased in the leaves of salt-tolerant cultivars acting as osmopotectants, but not in sensitive ones [2]. In addition, complex metabolic analysis revealed that there is only a partial overlap between the stress-signalling cascades that mediate proline and sugar accumulation [48], and that the metabolites involved in improved salt tolerance may differ in the roots and shoots [5,49].

The changes of starch content and the activity of the invertase enzyme revealed (Figs 7 and 8) that the elevated osmolyte accumulation in the leaves is only partially due to starch hydrolysis which produces maltose, and to an increase in invertase activity, which produces glucose and fructose from sucrose. The retention of CO_2_ assimilation capacity (S3 Table) in salt-tolerant genotypes could also be attributed to the *de novo* synthesis of assimilates such as glucose and galactose. Besides the glucose, fructose and sucrose that generally accumulate in salt-treated plants, trehalose and several oligosaccharides in the raffinose family, such as raffinose and stachyose, are also reported to be responsible for improved salt tolerance [48, 50].

Protection against salt-induced oxidative damage through an elevated antioxidant capacity could be an additional defensive strategy in plants, as demonstrated in comparative studies on cultivated and wild tomato species [51,52]. In the present experiment, however, although the activity of GR, APX and catalase increased in salt-treated plants, only the catalase activity was higher in salt-tolerant genotypes (barley cv. Manas, 7H and 7HL) than in wheat cv. Asakaze and addition line 7HS, especially in the roots (S4 Table). This in itself is unlikely to provide sufficient protection against salt-induced oxidative damage.

## Conclusions

It was demonstrated [14] in a recent study that the elevated salt tolerance of barley cv. Manas was manifested in wheat/barley addition lines 7H and 7HL. Osmotic potential measurements also suggested that osmotic adjustment might make an important contribution to salt tolerance in these genotypes.

**Fig 10:**
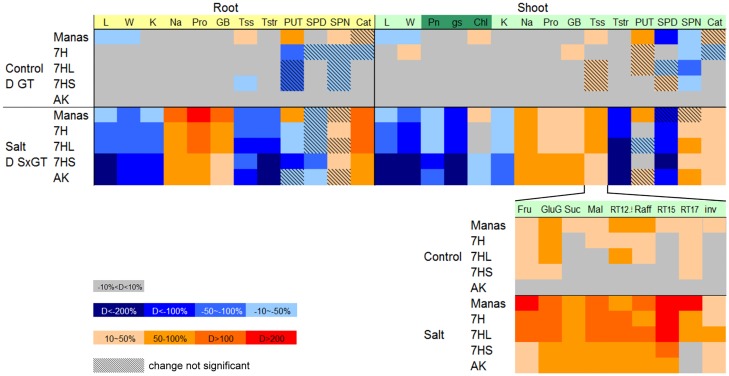
Comparison of growth, physiological traits and metabolic changes in the roots and shoots of different genotypes (wheat/barley disomic addition line 7H, ditelosomic addition lines 7HL and 7HS, wheat cv. Asakaze: AKand barley cv. Manas) under control and salt stress conditions. Comparisons were made for genotypes vs wheat cv. Asakaze in control plants, and salt-treated vs control plants. Grey blocks indicate values similar (within 10%) to the control values of wheat cv. Asakaze. Reddish and blue blocks indicate values higher and lower than those of wheat cv. Asakaze, respectively. L, length; W, weight; Pn, net photosynthetic rate; gs, stomatal conductance; Chl, chlorophyll content; Na and K, Na and K contents; Pro, proline; GB, glycine-betaine; Tss, total soluble sugars; Tstr, starch content; PUT, putrescine; SPD, spermidine, SPN, spermine; Cat, activity of catalase. Sugar compounds and invertase activity are presented separately for the leaves.

In the present work it was also shown that the salt response of the 7H disomic and 7HL ditelosomic addition lines differed from that of the wheat parent and the 7HS ditelosomic addition line. Furthermore, various physiological and biochemical processes and genes were tested as possible reasons for the improved salt tolerance. Differences between the genotypes (relative to the wheat parent Asakaze) and between their salt stress responses, based on all the parameters measured, are summarized in Fig 10.

Without salt stress few differences were found between the genotypes, such as the lower root and shoot growth of barley cv. Manas, elevated sugar content in the leaves of Manas and the addition lines, and modifications in PA contents in both the roots and shoots, while salt stress induced a complex metabolic rearrangement in both the roots and the shoots of all the genotypes. In the roots, Na uptake was accompanied by proline and GB accumulation, and an increase in catalase activity, especially in salt-tolerant cultivars (indicated by orange and red colours), while the amount of soluble sugars and starch decreased intensively, as indicated by the blue colour. In contrast, the sugar metabolism was the dominant process in the shoot. Higher quantities of sugars such as fructose, glucose+galactose and raffinose were found even in the control plants, and several sugars accumulated more intensively under salt stress in barley cv. Manas and addition lines 7H and 7HL than in addition line 7HS and wheat cv. Asakaze. These results suggest that osmotic adjustment is a more important process in the improvement of the salt tolerance in 7H addition lines than the direct regulation of Na transport processes or antioxidant defence. Barley chromosome 7HL may contain genes that positively affect the osmotic adjustment potential of wheat/barley addition lines under salt stress conditions.

## Supporting information

S1 Fig Discrimination of barley (red) and wheat (blue) mitotic chromosomes by Genomic *In Situ* Hybridization (GISH) in 7H Asakaze/Manas disomic addition (partial cell) (a), 7HS Asakaze/Manas ditelosomic addition (b) and 7HL Asakaze/Manas ditelosomic addition (c) lines. For method of GISH labelling see in [22].
(TIF)

S2 Fig Ct values of CDC(a) and RIL(a) genes when expressed in control and salt-treated leaves. (TIF)

S3 Fig K/Na ratio in the roots and shoots of different genotypes (wheat/barley disomic addition line 7H, ditelosomic addition lines 7HL and 7HS, wheat cv. Asakaze: AK and barley cv. Manas). (TIF)

S4 Fig Total soluble sugar content in the leaves of different genotypes (wheat/barley disomic addition line 7H, ditelosomic addition lines 7HL and 7HS, wheat cv. Asakaze: AK and barley cv. Manas) grown in hydroponic solution with or without salt treatment. Data are means ± SD of five replicates per treatment. Different letters indicate significant differences between the genotypes at P < 0.05 using Tukey’s *post hoc* test.
(TIF)

S5 Fig Conjugated Polyamine (PA) content in the roots and shoots of different genotypes (wheat/barley disomic addition line 7H, ditelosomic addition lines 7HL and 7HS, wheat cv. Asakaze: AK and barley cv. Manas) grown in hydroponic solution with and without salt treatment. Data are means ± SD of three replicates per treatment. * and # indicate values significantly different from the corresponding AK or untreated samples at the P < 0.05 level.
(TIF)

S1 Table List of gene-specific PCR primers used in the study. (DOC)

S2 Table Root and shoot length of wheat/barley addition lines 7H, 7HL and 7HS and their wheat cv. Asakaze and barley cv. Manas parents before salt treatment and at the end of the experiment with (salt-treated) or without (control) salt application. (DOC)

S3 Table Photosynthetic activity, stomatal conductance and SPAD chlorophyll content in wheat cv. Asakaze, barley cv. Manas and addition lines 7H, 7HL and 7HS. (DOC)

S4 Table Activity of the antioxidant enzymes Glutathione Reductase (GR), ascorbate reductase (APX), Monodehydroascorbate Reductase (MDHAR), catalase and Glutathione-S-Transferase (GST) in the roots and leaves of wheat cv. Asakaze: AK, barley cv. Manas and addition lines 7H, 7HL and 7HS. (DOC)
