# Supplementary figures and images for: Differing metabolic responses to salt stress in wheat-barley addition lines containing different 7H chromosomal fragments
Source: PLoS One. 2017 Mar 22;12(3):e0174170. doi: 10.1371/journal.pone.0174170 (PMC5362201; doi:10.1371/journal.pone.0174170)

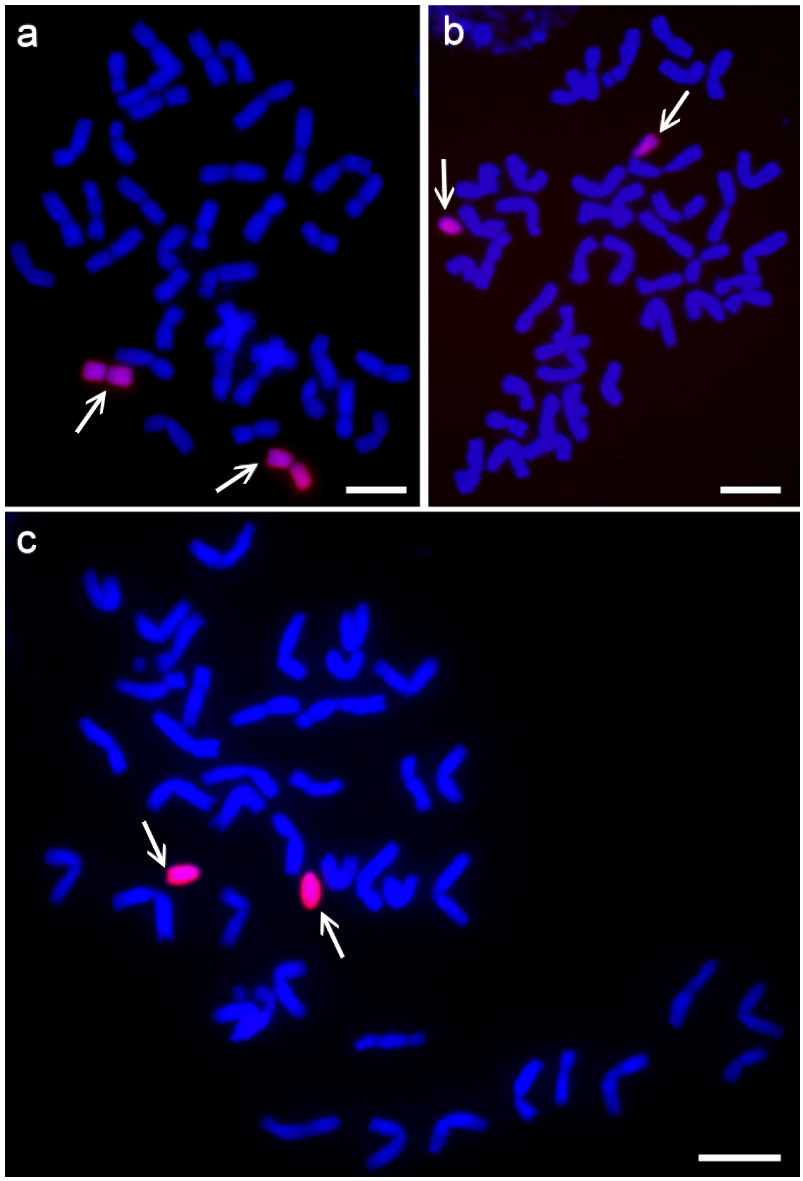

Supplement: S1 Fig — For method of GISH labelling see in [22]. (TIF) [file pone.0174170.s001.tif]

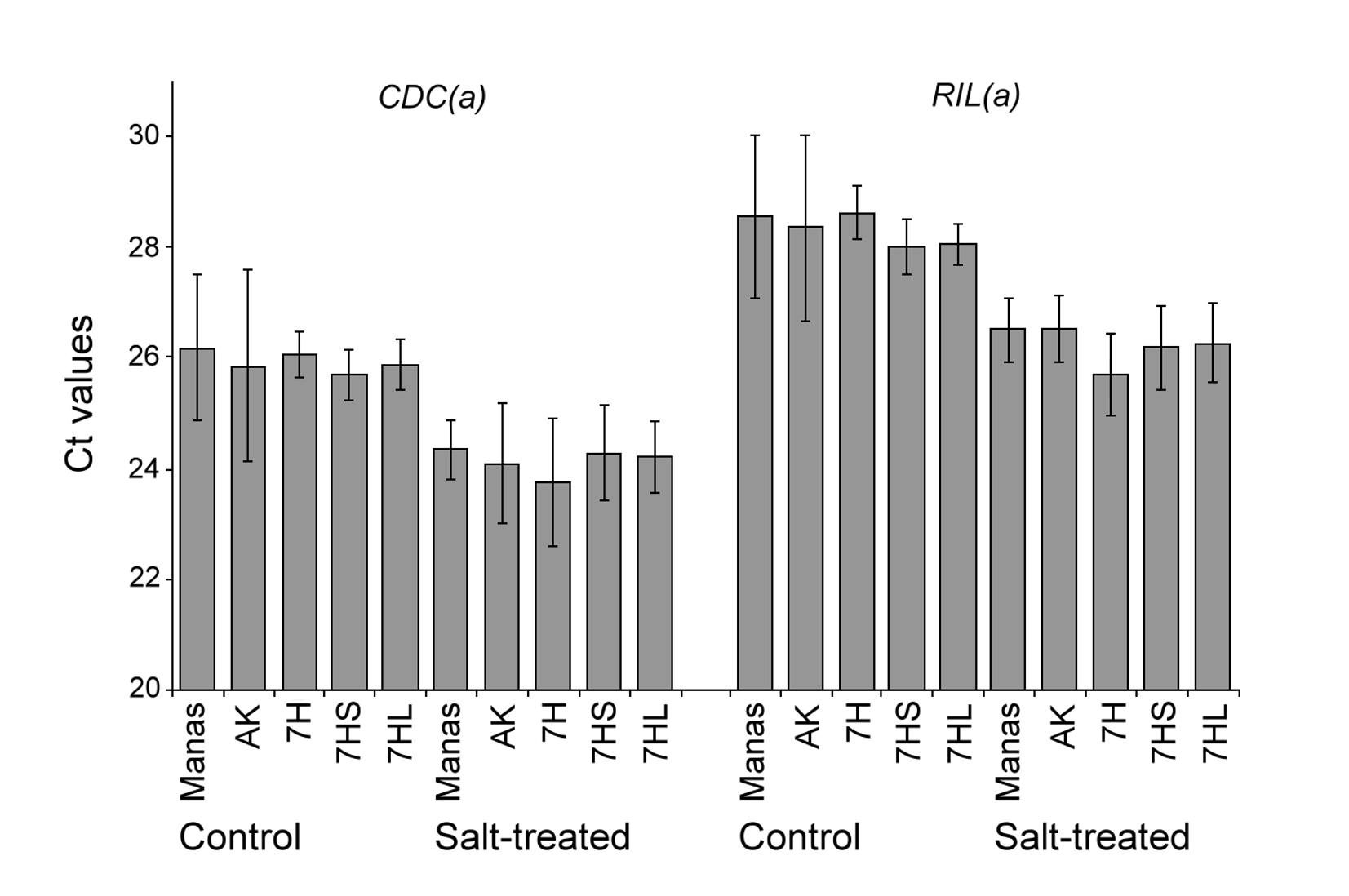

Supplement: S2 Fig — (TIF) [file pone.0174170.s002.tif]

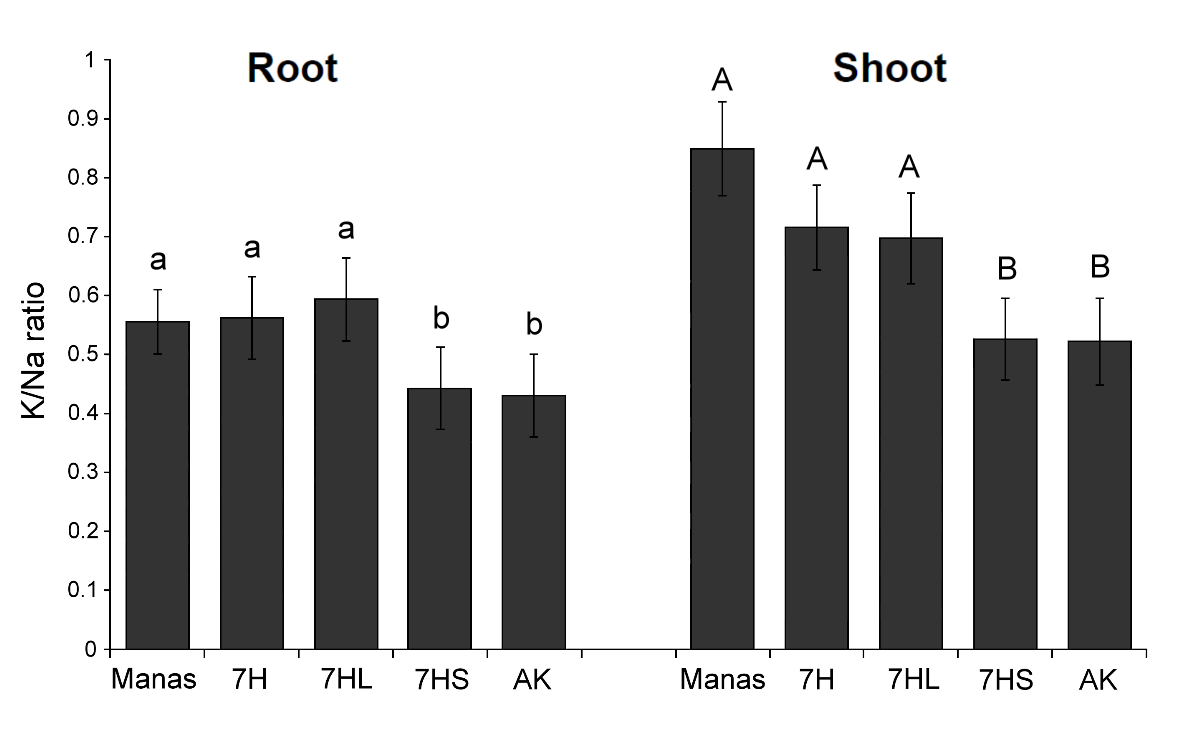

Supplement: S3 Fig — (TIF) [file pone.0174170.s003.tif]

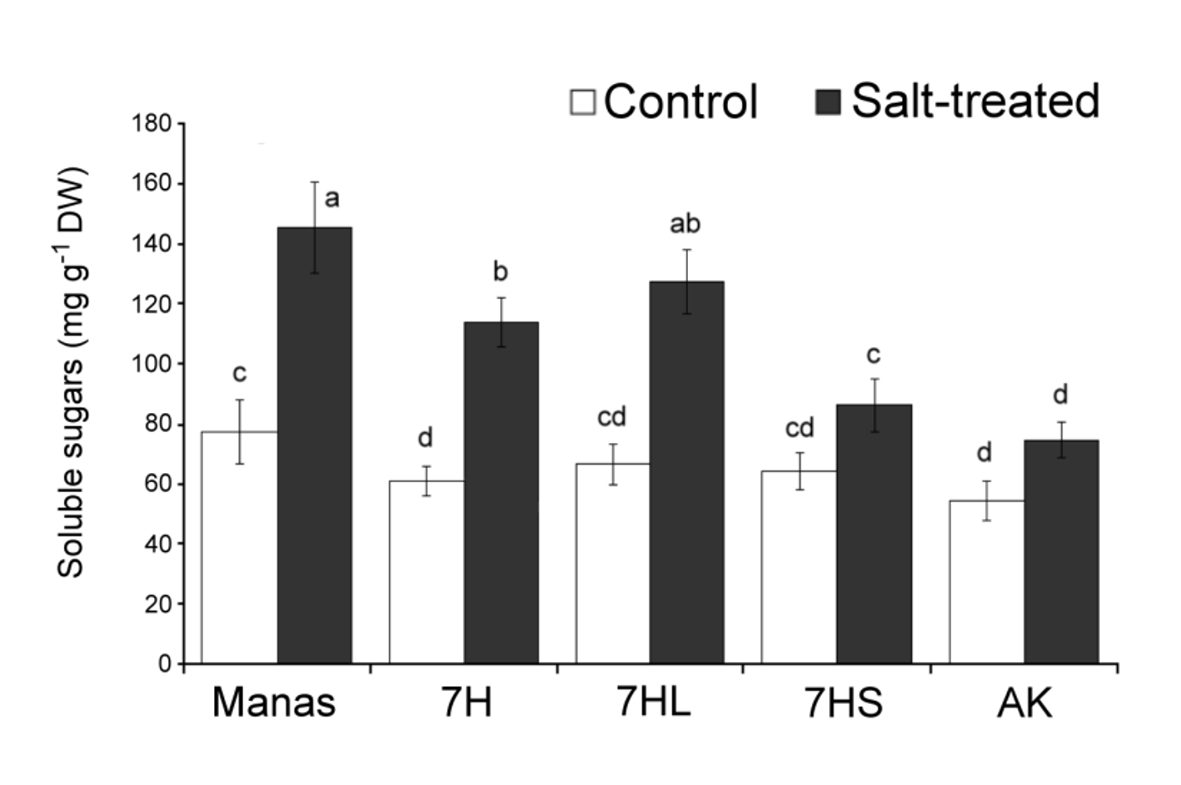

Supplement: S4 Fig — Data are means ± SD of five replicates per treatment. Different letters indicate significant differences between the genotypes at P < 0.05 using Tukey’s post hoc test. (TIF) [file pone.0174170.s004.tif]

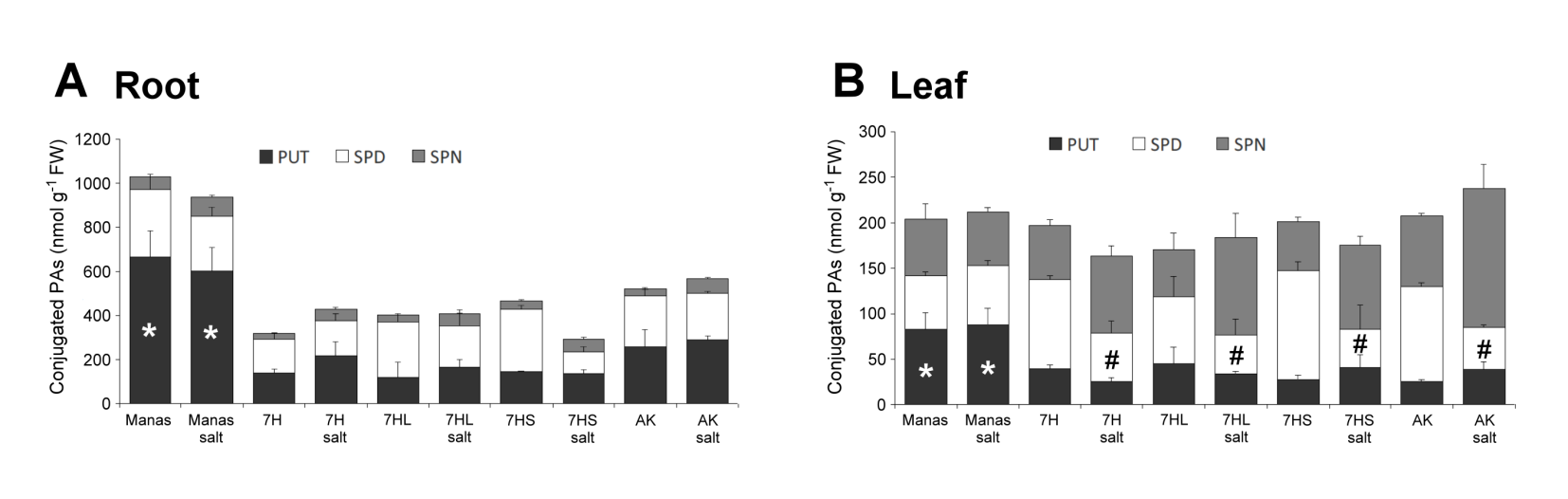

Supplement: S5 Fig — Data are means ± SD of three replicates per treatment. * and # indicate values significantly different from the corresponding AK or untreated samples at the P < 0.05 level. (TIF) [file pone.0174170.s005.tif]
