# Supplementary material for: Differing metabolic responses to salt stress in wheat-barley addition lines containing different 7H chromosomal fragments
Source: PLoS One. 2017 Mar 22;12(3):e0174170. doi: 10.1371/journal.pone.0174170 (PMC5362201; doi:10.1371/journal.pone.0174170)
Supplement: S1 Table — (DOC) [file pone.0174170.s006.doc]

**S1** Table: List of gene-specific PCR primers used in the study.

| **Name of gene** | **Primer sequences (5' → 3')** | | **Gene ID** | **Reference** |
| --- | --- | --- | --- | --- |
| *Hv Cyclophilin* | Forward | CCTGTCGTGTCGTCGGTCTAAA | AK253120.1 | [21] |
|  | Reverse | ACGCAGATCCAGCAGCCTAAAG |  |  |
| *Ta 30797* | Forward | GCCGTGTCCATGCCAGTG | TC279294 TC284282 | [22] |
| *(Similar to phosphogluconate dehydrogenase)* | Reverse | TTAGCCTGAACCACCTGTGC |  |  |
| *CDC(a)* | Forward | CAGCTGCTGACTGAGATGGA | Ta54227 | [23] |
| *(Cell division control protein)* | Reverse | ATGTCTGGCCTGTTGGTAGC |  |  |
| *RLI(a)* | Forward | TTGAGCAACTCATGGACCAG | Ta2776 | [23] |
| *(Similar to A. thaliana RNaseL inhibitor protein)* | Reverse | GCTTTCCAAGGCACAAACAT |  |  |
| *SOS1* | Forward | CATGCTGGGAGAGTCCACT | KJ563230.1 MLOC_36509 | own design |
| *(Salt overly sensitive 1)* | Reverse | ACACGCGGCCTCTGCTCT |  |  |
| *SOS2* | Forward | GAAAACCTGCTTCTTGATTCACG | JP218831.1 JN655679 | own design |
| *(Salt overly sensitive 2)* | Reverse | GCTGCAGATCCATCATAGCC |  |  |
| *HKT1* | Forward | TGTCCTACTTCCTTGCCATTGC | AM000056.1 U16709.1 | own design |
| *(High-affinity K^+^ transporter)* | Reverse | TCGTGATGGTGCTGAGGC |  |  |
| *NHX2* | Forward | AATAAGCTGGAGGCAGCAAA | AY040246.2 AB089197.1 | own design |
| *(Na^+^/H^+^ antiporter)* | Reverse | GTGCTAAACAGAACGACAGT |  |  |
| *HVP1* | Forward | AAAGAGCCTGGGCCCGAAAGGC | AB032839.1 | [7] |
| *(Vacuolar H^+^-pyrophosphatase)* | Reverse | TCTTGAAGAGGATTCCTCCATAG |  |  |
