# Supplementary material for: Differing metabolic responses to salt stress in wheat-barley addition lines containing different 7H chromosomal fragments
Source: PLoS One. 2017 Mar 22;12(3):e0174170. doi: 10.1371/journal.pone.0174170 (PMC5362201; doi:10.1371/journal.pone.0174170)
Supplement: S2 Table — (DOC) [file pone.0174170.s007.doc]

**S2 Table. Root and shoot length of wheat/barley disomic addition line 7H, ditelosomic addition lines 7HL and 7HS, and their wheat cv. Asakaze (AK) and barley cv. Manas parents before salt treatment and at the end of the experiment with (salt-treated) or without (control) salt application.**

|  | Root length (cm) | | | Shoot length (cm) | | |
| --- | --- | --- | --- | --- | --- | --- |
|  | Before treatment | Control | Salt-treated | Before treatment | Control | Salt-treated |
| Manas | 7.3±0.54  f | 16.6±0.60  b | 13.4±0.74  cd (65%) | 11.53±0.93  f | 23.04±1.03  b | 20.4±0.97  c (77%) |
| AK/Manas 7H | 11.3±0.63  e | 21.0±0.61  a | 16.8±0.54  b (56%) | 15.96±1.06  e | 26.94±1.13  a | 23.8±0.91  b (71%) |
| AK/Manas 7HL | 10.5±0.61  e | 21.9±0.69  a | 17.4±0.65  b (60%) | 15.25±1.15  e | 26.75±1.26  a | 23.0±1.17  b (67%) |
| AK/Manas 7HS | 10.6±0.52  e | 22.0±0.56  a | 12.8±0.50  d (20%) | 15.84±1.03  e | 25.6±1.16  a | 18.72±1.09  d (29%) |
| AK | 10.9±0.68  e | 21.9±0.62  a | 14.3±0.66  c (31%) | 15.43±0.98  e | 25.0±1.22  ab | 17.66±0.93  d (23%) |

Salt-induced growth reduction was indicated as a % of the control, calulated as (Salt-treated – Before treatment)/(Control – Before treatment) *100. Data are means ± SD of 25 replicates per treatment and genotype. Different letters indicate significantly differences between the genotypes at P < 0.05 using Tukey’s *post hoc* test.
