# Supplementary material for: Differing metabolic responses to salt stress in wheat-barley addition lines containing different 7H chromosomal fragments
Source: PLoS One. 2017 Mar 22;12(3):e0174170. doi: 10.1371/journal.pone.0174170 (PMC5362201; doi:10.1371/journal.pone.0174170)
Supplement: S3 Table — (DOC) [file pone.0174170.s008.doc]

**S3 Table.** Photosynthetic activity (Pn), stomatal conductance (gs) and SPAD chlorophyll content measured at the end of the experiments in wheat cv. Asakaze, barley cv. Manas and addition lines 7H, 7HL and 7HS.

|  | Pn | | gs | | SPAD values | |
| --- | --- | --- | --- | --- | --- | --- |
|  | Control | 200mM NaCl | Control | 200 mM NaCl | Control | 200 mM NaCl |
| Manas | 14.6±0.76  a | 11.6±0.69  (79%) c | 346±18.8  a | 154±12  (45%) b | 47.6±2.6  a | 42.3±3.2  ab |
| AK/Manas 7H | 14.8±0.69  e | 12.4±0.79  (84%) bc | 322±15.2  a | 145±9.6  (45%) b | 42.0±2.5  b | 37.7±2.8  b |
| AK/Manas 7HL | 14.6±0.72  a | 12.2±0.67  (83%) c | 334±14.6  a | 138±10  (41%) b | 43.3±2.7  ab | 40.3±2.0  b |
| AK/Manas 7HS | 13.8±0.90  ab | 6.2±0.75  (45%) e | 311±23.1  a | 65±11  (21%) d | 41.62±2.2  b | 31.64±2.2  c |
| AK | 14.4±0.89  a | 8.8±0.70  (61%) d | 332±1341  a | 92±15  (28%) c | 40.6±2.6  b | 31.7±2.6  c |

Values are means ± SD of five replicates for Pn and gs and 25 for SPAD values determined at the end of the experiments. Different letters indicate significant differences between the genotypes at P < 0.05 using Tukey’s *post hoc* test.
