# Supplementary material for: Differing metabolic responses to salt stress in wheat-barley addition lines containing different 7H chromosomal fragments
Source: PLoS One. 2017 Mar 22;12(3):e0174170. doi: 10.1371/journal.pone.0174170 (PMC5362201; doi:10.1371/journal.pone.0174170)
Supplement: S4 Table — (DOC) [file pone.0174170.s009.doc]

**S4** Table Activity of the antioxidant enzymes glutathione reductase (GR), ascorbate reductase (APX), monodehydroascorbate reductase (MDHAR), catalase and glutathione-S-transferase (GST) in the roots and leaves of wheat cv. Asakaze: AK, barley cv. Manas and addition lines 7H, 7HL and 7HS.

|  | | GR | | APX | | DHAR | | catalase | | GST | |
| --- | --- | --- | --- | --- | --- | --- | --- | --- | --- | --- | --- |
|  |  | Control | Salt-treated | Control | Salt-treated | Control | Salt-treated | Control | Salt-treated | Control | Salt-treated |
| R  o  o  t | AK | 0.337±0.054  b | 0.508±0.062  a | 18.94±1.64  bc | 23.62±1.54  a | 2.33±0.34  ab | 1.75±0.21  c | 25.8±3.4  c | 45.4±7.2  b | 2.13±0.25  ab | 2.38±0.29  a |
|  | 7HS | 0.249±0.048  c | 0.436±0.069  a | 18.10±1.96  bc | 22.98±1.65  a | 2.21±0.24  b | 1.67±0.20  c | 22.9±3.6  c | 55.2±6.5  b | 1.86±0.19  b | 1.78±0.16  b |
|  | 7H | 0.257±0.039  c | 0.398±0.056  ab | 16.98±2.08  c | 23.33±2.49  a | 2.14±0.27  b | 2.04±0.22  bc | 21.6±4.2  c | 68.7±7.4  a | 1.89±0.23  b | 2.01±0.21  ab |
|  | 7HL | 0.212±0.042  c | 0.433±0.068  a | 20.34±1.96  ab | 22.92±1.87  a | 2.36±0.26  ab | 1.94±0.23  bc | 25.6±3.9  c | 82.4±7.9  a | 1.93±0.24  b | 1.84±0.26  b |
|  | Manas | 0.3483±0.045  b | 0.482±0.065  a | 20.42±1.75  ab | 24.93±1.93  a | 2.87±0.35  a | 2.50±0.36  ab | 31.3±4.6  c | 78.0±8.5  a | 1.974±0.25  b | 2.46±0.27  a |
|  |  |  |  |  |  |  |  |  |  |  |  |
| L  e  a  f | AK | 1.17±0.154  c | 1.61±0.100  ab | 21.09±2.73  a | 22.33±1.49  a | 3.26±0.35  cd | 3.96±0.29  b | 748±89  c | 931±92  b | 2.76±0.32  ab | 3.27±0.25  a |
|  | 7HS | 0.93±0.109  cd | 1.42±0.104  b | 22.05±2.85  a | 23.01±1.85  a | 3.82±0.41  bc | 4.72±0.25  a | 725±82  c | 920±87  b | 3.23±0.33  a | 3.26±0.28  a |
|  | 7H | 0.86±0.106  d | 1.43±0.177  b | 21.89±2.91  a | 23.75±2.5  a | 3.12±0.42  cd | 3.82±0.47  bc | 731±75  c | 1032±112  ab | 2.71±0.21  b | 3.32±0.26  a |
|  | 7HL | 1.14±0.105  c | 1.79±0.106  a | 20.64±1.57  a | 22.49±1.61  a | 2.79±0.33  d | 3.85±0.35  b | 759±82  c | 1056±104  ab | 3.04±0.18  ab | 3.13±0.31  a |
|  | Manas | 1.02±0.107  cd | 1.42±0.105  b | 19.82±1.46  a | 20.71±1.83  a | 3.85±0.59  bc | 4.3±0.42  ab | 888±95  bc | 1169±111  a | 2.05±0.19  c | 2.52±0.38  bc |
|  |  |  |  |  |  |  |  |  |  |  |  |

Activities are expressed in μmol substrate min^-1^ g^-1^ FW. Data represent mean values ± SD of five replicates per treatment. Different letters indicate significant differences between the genotypes at P < 0.05 using Tukey’s *post hoc* test.
